# Simplified Epigenome Profiling Using Antibody-tethered Tagmentation

**DOI:** 10.21769/BioProtoc.4043

**Published:** 2021-06-05

**Authors:** Steven Henikoff, Jorja G. Henikoff, Kami Ahmad

**Affiliations:** 1 Basic Sciences Division, Fred Hutchinson Cancer Research Center, 1100 Fairview Ave N, Seattle, Washington 98109, USA; 2 Howard Hughes Medical Institute, Seattle WA, USA

**Keywords:** Epigenomic profiling, Chromatin accessibility, RNA polymerase II, Histone modifications, CUT&Tag

## Abstract

We previously introduced Cleavage Under Targets & Tagmentation (CUT&Tag), an epigenomic profiling method in which antibody tethering of the Tn5 transposase to a chromatin epitope of interest maps specific chromatin features in small samples and single cells. With CUT&Tag, intact cells or nuclei are permeabilized, followed by successive addition of a primary antibody, a secondary antibody, and a chimeric Protein A-Transposase fusion protein that binds to the antibody. Addition of Mg^++^ activates the transposase and inserts sequencing adapters into adjacent DNA *in situ*. We have since adapted CUT&Tag to also map chromatin accessibility by simply modifying the transposase activation conditions when using histone H3K4me2, H3K4me3, or Serine-5-phosphorylated RNA Polymerase II antibodies. Using these antibodies, we redirect the tagmentation of accessible DNA sites to produce chromatin accessibility maps with exceptionally high signal-to-noise and resolution. All steps from nuclei to amplified sequencing-ready libraries are performed in single PCR tubes using non-toxic reagents and inexpensive equipment, making our simplified strategy for simultaneous chromatin profiling and accessibility mapping suitable for the lab, home workbench, or classroom.

## Background

Mapping of DNA accessibility in the chromatin landscape was first described 45 years ago with the observation of DNaseI hypersensitivity at transcriptionally active loci (Weintraub and Groudine, 1976). Because DNaseI preferentially cleaves genomic regions that are depleted of nucleosomes, and regulatory elements are bound by non-histone chromatin proteins rather than nucleosomes, DNaseI hypersensitive site mapping has since been used to characterize the genetic regulatory landscape. Other enzymatic probes of chromatin accessibility include Micrococcal Nuclease (MNase) (Reeves *et al.*, 1978), restriction endonucleases (Jack and Eggert, 1990), transposases (Bownes, 1990), and DNA methyltransferases (Gottschling *et al.*, 1992). Hypersensitive site mapping became more routine with the introduction of genome-wide read-out platforms, beginning with microarrays and later short-read DNA sequencing (Crawford *et al.*, 2004; Dorschner *et al.*, 2004). Chromatin accessibility was also mapped using physical fragmentation and differential recovery of cross-linked chromatin, the basis for FAIRE (Giresi *et al.*, 2007) and Sono-Seq (Auerbach *et al.*, 2009). In recent years, the most popular chromatin accessibility mapping method has been ATAC-seq (Buenrostro *et al.*, 2013), in which the Transposon 5 (Tn5) cut-and-paste transposition reaction inserts sequencing adapters in the most accessible genomic regions (tagmentation). Because tagmentation creates sequencing libraries simultaneous with insertion into accessible sites, ATAC-seq is simple and fast, and successively improved ATAC-seq protocols have enhanced its popularity (Corces *et al.*, 2016 and 2017).

Despite the utility of chromatin accessibility mapping, the mechanistic basis for chromatin accessibility itself has remained incompletely understood. In contrast to the simplistic designation of chromatin as being “open” or “closed,” recent work has shown that the median difference between an accessible and a non-accessible site in DNA is estimated to be only ~20%, with no sites completely accessible or inaccessible in a population of cells (Chereji *et al.*, 2019; Oberbeckmann *et al.*, 2019). To better understand this nuanced interpretation of chromatin accessibility, we have recently applied our Cleavage Under Targets & Tagmentation (CUT&Tag) method for antibody-tethered *in situ* tagmentation of chromatin to explore the mechanistic basis for chromatin accessibility (Henikoff *et al.*, 2020). CUT&Tag uses a fusion protein between Protein A, which binds to the chromatin-bound antibody, and Tn5, which binds to adjacent DNA, and tagmentation occurs upon activation with Mg^++^ (Kaya-Okur *et al.*, 2019). To suppress artifactual tagmentation of untargeted accessible chromatin, we performed all steps from pA-Tn5 fusion protein binding through tagmentation in the presence of 300 mM NaCl, which reduces non-specific DNA binding of the transposase. In the course of optimizing a simplified single-tube protocol, CUT&Tag-direct (Kaya-Okur *et al.*, 2020), we serendipitously observed that simply reducing the ionic concentration during antibody-targeted tagmentation greatly increased the tendency of tethered Tn5 to tagment accessible chromatin near particular histone modifications (Henikoff *et al.*, 2020). Preferential tagmentation of accessible chromatin only occurred when using antibodies against H3K4me2 and H3K4me3 and not for other histone modifications or variants. Because H3K4me2 flanks both promoters and enhancers genome-wide, the attraction of antibody-tethered Tn5 to nearby accessible DNA regions shifted the preferred sites of tagmentation from the nucleosomes bordering the Nucleosome-Depleted Region (NDR) to the NDR itself. Remarkably, practically all transcription-coupled accessible sites corresponded to ATAC-seq sites and vice-versa, upstream of paused RNA Polymerase II (RNAPII). Because of the close correspondence between the resulting “CUTAC” (Cleavage Under Targeted Accessible Chromatin) maps and DNaseI and ATAC-seq chromatin accessibility maps, we concluded that chromatin accessibility is driven by RNAPII transcriptional initiation (Henikoff *et al.*, 2020), supporting previous suggestions that active promoters and enhancers are characterized by the same regulatory architecture (Andersson *et al.*, 2015; Arnold *et al.*, 2019).

In our original CUTAC study, we described three different modifications of the CUT&Tag-direct protocol for accessible site mapping: tagmentation in MgCl_2_ with a 20-fold dilution of 300 mM NaCl and pA-Tn5 (or commercial pAG-Tn5 with both Protein A and Protein G IgG specificities), removal of excess pAG-Tn5 before low-salt tagmentation, and low-salt tagmentation following the 300 mM wash step. We have adopted post-wash tagmentation, which follows the same steps as in the original CUT&Tag-direct protocol (Kaya-Okur *et al.*, 2020), changing only the tagmentation buffer composition. As reported here, the application of this CUTAC protocol to the initiation form of RNAPII results in precise chromatin accessibility maps with exceptionally high signal-to-noise. The improvement obtained by tethering to the transcriptional machinery itself further supports the transcription-coupled basis for chromatin accessibility at enhancers and promoters.

CUT&Tag and CUTAC can be performed simultaneously in a single day from previously frozen native or lightly cross-linked nuclei through to purified sequencing-ready libraries, with all steps carried out in single PCR tubes. We present a simplified protocol where all steps from nuclei to purified sequencing-ready libraries are performed on a home benchtop using surplus equipment and non-toxic reagents (Figure 1). Our CUTAC results using an antibody to the Serine-5-phosphorylated initiation form of the repeated heptameric C-terminal domain of the largest RNAPII subunit (RNAPIIS5P) compare favorably with the best ATAC-seq data while providing a genome-wide map of the initiation form of RNAPII. The simplicity and affordability of the protocol make it equally suitable for a laboratory, home, or classroom environment.

## Materials and Reagents

1. Disposable tips (*e.g.*, Rainin 1 ml, 200 µl, 20 µl)
2. Disposable centrifuge tubes for reagents (15 ml or 50 ml)
3. Standard 1.5 ml microfuge tubes
4. 0.5 ml maximum recovery PCR tubes (*e.g.*, Fisher, catalog number: 14-222-294)
5. Phosphate-buffered saline (Fisher cat. no. BP3994)
6. 16% (w/v) formaldehyde (10 × 1 ml ampules, Thermo Fisher, catalog number: 28906)
7. 1.25 M glycine (Sigma-Aldrich, catalog number: G7126)
8. Dimethyl sulfoxide (DMSO; Sigma-Aldrich, catalog number: D4540)
9. Cell culture (*e.g.*, human K562 cells)
10. Concanavalin A (ConA)-coated magnetic beads (Bangs Laboratories, catalog number: BP531)
11. Distilled, deionized, or RNAse-free H_2_O (dH_2_O; *e.g.*, Promega, catalog number: P1197)
12. 1 M Hydroxyethyl piperazineethanesulfonic acid pH 7.9 (HEPES (K^+^); Sigma-Aldrich, catalog number: H3375)
13. 1 M Manganese Chloride (MnCl_2_; Sigma-Aldrich, catalog number: 203734)
14. 1 M Calcium Chloride (CaCl_2_; Fisher, catalog number: BP510)
15. 1 M Potassium Chloride (KCl; Sigma-Aldrich, catalog number: P3911)
16. Roche Complete Protease Inhibitor EDTA-Free tablets (Sigma-Aldrich, catalog number: 5056489001)
17. 1 M Hydroxyethyl piperazineethanesulfonic acid pH 7.5 (HEPES (Na^+^); Sigma-Aldrich, catalog number: H3375)
18. 5 M Sodium chloride (NaCl; Sigma-Aldrich, catalog number: S5150-1L)
19. 2 M Spermidine (Sigma-Aldrich, catalog number: S0266)
20. 0.5 M Ethylenediaminetetraacetic acid (EDTA; Research Organics, catalog number: 3002E)
21. 200x Bovine Serum Albumen (BSA; NEB, catalog number: B9001S)
22. Antibody to an epitope of interest for CUT&Tag
Because *in situ* binding conditions are more like those for immunofluorescence (IF) than those for ChIP, we suggest choosing IF-tested antibodies if CUT&RUN/Tag-tested antibodies are not available.
23. CUTAC control antibody to RNA Polymerase II Phospho-Rpb1 CTD Serine-5 phosphate (RNAPIIS5P) or histone H3K4me2. We have obtained excellent results with these rabbit monoclonal antibodies:
  Phospho-Rpb1 CTD (Ser5) (Cell Signalling Technology, catalog number: 13523 (D9N5I))
  H3K4me2 (Epicypher, catalog number: 13-0027)
24. Secondary antibody, *e.g.*, guinea pig α-rabbit antibody (Antibodies-Online, catalog number: ABIN101961) or rabbit α-mouse antibody (Abcam, catalog number: ab46540)
25. Protein A/G-Tn5 (pAG-Tn5) fusion protein loaded with double-stranded adapters with 19mer Tn5 mosaic ends (Epicypher, catalog number: 15-1117)
26. 1 M Magnesium Chloride (MgCl_2_; Sigma-Aldrich, catalog number: M8266-100G)
27. 1 M [tris(hydroxymethyl)methylamino] propanesulfonic acid (TAPS) pH 8.5 (with NaOH)
28. 1,6-hexanediol (Sigma-Aldrich, catalog number: 240117-50G)
29. N,N-dimethylformamide (Sigma-Aldrich, catalog number: D-8654-250 ml)
30. NEBNext 2× PCR Master mix (ME541L)
31. PCR primers: 10 µM stock solutions of i5 and i7 primers with unique barcodes [Buenrostro, J.D. *et al.*, Nature 523:486 (2015)] in 10 mM Tris pH 8. Standard salt-free primers may be used. We do not recommend Nextera or NEBNext primers.
32. 10% Sodium dodecyl sulfate (SDS; Sigma-Aldrich, catalog number: L4509)
33. 10% Triton X-100 (Sigma-Aldrich, catalog number: X100)
34. SPRI paramagnetic beads (*e.g.*, HighPrep PCR Cleanup Magbio Genomics, catalog number: AC-60500)
35. 10 mM Tris-HCl pH 8.0
36. Ethanol (Decon Labs, catalog number: 2716)
37. Nuclei Extraction 1 (NE1) buffer (see Recipes)
38. Wash buffer (see Recipes)
39. Binding buffer (see Recipes)
40. Antibody buffer (see Recipes)
41. 300-wash buffer (see Recipes)
42. CUTAC Tagmentation buffer (see Recipes)
43. TAPS wash buffer (see Recipes)
44. 0.1% SDS Release solution (see Recipes)
45. 0.67% Triton neutralization solution (see Recipes)

## Equipment

-80°C freezer
2. Chilling device (*e.g.*, metal heat blocks on ice or cold packs in an ice cooler)
3. Pipettors (*e.g.*, Rainin Classic Pipette 1 ml, 200 µl, 20 µl, and 10 µl)
4. Strong magnet stand (*e.g.*, Miltenyi Macsimag separator, catalog number: 130-092-168)
5. Vortex mixer (*e.g.*, VWR Vortex Genie)
6. Minicentrifuge (*e.g.*, VWR Model V)
7. Tube rotator (*e.g.*, Barnstead/Thermolyne 400110)
8. PCR thermocycler (*e.g.*, Bio-Rad/MJ PTC-200)
  **Figure 1.**
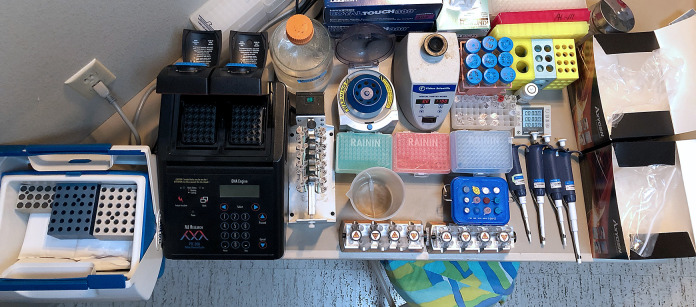
A home workbench for CUT&Tag. Photo of the home workbench setup used for all experiments presented using this protocol. A typical experiment begins by mixing cells with activated ConA beads in up to 32 single PCR tubes, with all liquid changes performed on the magnet stand. The only tube transfer is the removal of the purified sequencing-ready libraries from the SPRI beads to fresh tubes for Tapestation analysis and DNA sequencing. The total time from thawing frozen nuclei until elution from SPRI beads is ~8 h.

## Software

1. Bowtie2 http://bowtie-bio.sourceforge.net/bowtie2/index.shtml

2. Calibration script https://github.com/Henikoff/Cut-and-Run/blob/master/spike_in_calibration.csh

## Procedure

**Figure 2.**
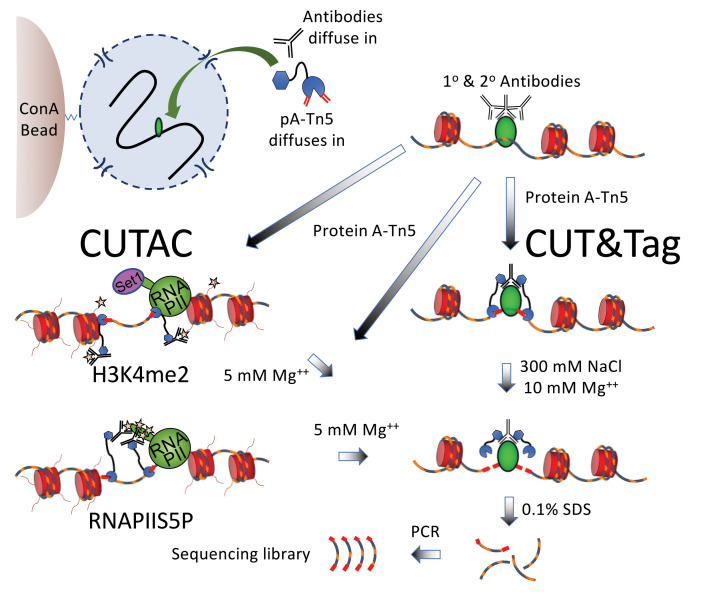
Scheme for simultaneous CUT&Tag and (H3K4me2 or RNAPIIS5P) CUTAC. CUT&Tag-direct is performed *in situ* in single PCR tubes with Concanavalin A (ConA) bead-bound nuclei that remain intact throughout the protocol during successive liquid changes, incubations and washes, 12 cycles of PCR amplification, and one SPRI bead cleanup. CUTAC is performed identically except that low-salt conditions are used for tagmentation. H3K4me2 CUTAC maps accessible sites near H3K4me2/3-marked (starred) nucleosome tails, which are methylated by the conserved Set1 lysine methyltransferase. The complex that includes Set1 associates with the initiation form of RNAPII, which is heavily phosphorylated on Serine-5 of the heptameric C-terminal domain repeat units on the largest RNAPII subunit (RNAPIIS5P). For RNAPIIS5P CUTAC, pA-Tn5 is anchored directly to RNAPIIS5 phosphates (starred). Whereas CUT&Tag is suitable for any chromatin epitope, CUTAC is specific for H3K4me2, H3K4me3, and RNAPIIS5P. The only other difference between the protocols is that tagmentation is performed in the presence of 300 mM NaCl for CUT&Tag and in a low ionic strength buffer for CUTAC.

- Prepare and (optionally) lightly fix nuclei and cryopreserve (1 h in the lab)
  1. Harvest fresh culture(s) in a conical centrifuge tube (15 ml or 50 ml) at room temperature and count cells.
  2. Centrifuge for 3 min at 600 *× g* in a swinging bucket rotor at room temperature and drain the liquid.
  3. Resuspend in 1 volume of PBS (relative to starting culture) at room temperature by pipetting up and down.
  4. Centrifuge for 3 min at 600 *× g* in a swinging bucket rotor at room temperature and drain the liquid.
  5. Resuspend in 1/2 volume (relative to starting culture) of ice-cold NE1 buffer with gentle vortexing. Let sit on ice for 10 min.
  6. Centrifuge for 4 min at 1,300 *× g* at 4°C in a swinging bucket rotor and drain liquid by pouring off and inverting onto a paper towel for a few seconds.
  7. Resuspend in 1/2 volume of PBS. For unfixed nuclei, skip to Step A11.
  8. While gently vortexing, add 16% formaldehyde to 0.1% (*e.g.*, 62 μl to 10 ml) and incubate at room temperature for 2 min.
*Note: Light fixation reduces the tendency of cells or nuclei to clump in the 300-wash buffer but can interfere with the binding of some antibodies, reducing yield.*
  9. Stop cross-linking by adding 1.25 M glycine to twice the molar concentration of formaldehyde (*e.g.*, 600 μl to 10 ml).
  10. Centrifuge for 4 min at 1,300 *× g* at 4°C and drain the liquid by pouring off and inverting onto a paper towel for a few seconds.
  11. Resuspend in Wash buffer to a concentration of ~1 million cells per ml. Check nuclei using ViCell or a cell counter slide.
  12. Nuclei may be slowly frozen by aliquoting 900 μl into cryogenic vials containing 100 μl of DMSO, mixed well, then placed in a Mr. Frosty container filled to the line with isopropanol and placed in a -80°C freezer overnight and stored at -80°C long term.
*Note: We have found that good results are obtained using native or cross-linked cells even after being stored in the freezer compartment of a side-by-side refrigerator for >6 months.*
- Prepare Concanavalin A-coated beads (15 min)
  1. Resuspend and withdraw enough of the ConA bead slurry, ensuring that there will be 3.5 μl for each final sample of up to ~50,000 mammalian cells, which yield ≥50% K562 nuclei using this protocol. Transfer the ConA bead slurry into 1 ml of Binding buffer in a 1.5 ml tube.
*Note: This protocol has been used for up to 16 samples (60 µl beads) in 1 ml or 32 samples (120 µl beads) in 2 ml Binding buffer (in a 2 ml tube).*
  2. Mix by pipetting. Place the tube on a magnet stand to clear (~1 min).
  3. Withdraw the supernatant completely and remove the tube from the magnet stand. Add 1 ml Binding buffer and mix by pipetting up and down.
  4. Place on the magnet stand to clear, remove and discard the supernatant, and resuspend in 60 µl Binding buffer (3.5 μl per sample).
- Bind nuclei to ConA beads (15 min)
  1. Thaw a frozen aliquot of nuclei at room temperature, for example, by placing in a 20 ml beaker of water.
*Note: The CUTAC control can use either native or lightly cross-linked nuclei, preferably prepared as previously described (Kaya-Okur et al., 2020). Do not use whole cells, which require a detergent and may also inhibit the PCR.*
  2. Transfer the thawed nuclei suspension in aliquots of no more than ~50,000 starting mammalian cells to each thin-wall 0.5 ml PCR tube and mix with 3.5 µl ConA beads. Attach to the Tube rotator and rotate at room temperature for 10 min.
*Note: Nuclei prepared according to the recommended protocol (Kaya-Okur et al., 2020) have been resuspended in Wash buffer. Beads can be added directly to the aliquot for binding and then transferred to PCR tubes, ensuring that no more than 5 µl of the original ConA bead suspension is present in each PCR tube for single-tube CUT&Tag. Using more than ~50,000 mammalian nuclei or >5 µl Con A beads per sample may inhibit the PCR.*
  3. Place the tubes on the magnet stand to clear and remove and discard the supernatant.
*Note: In low-retention PCR tubes, surface tension will cause bead-bound cells to slide down to the bottom of the tube at this step. To avoid beads being aspirated with the supernatant, set the pipette to a volume that is 5 µl less than the total volume to be removed. Use a careful second draw with a 20 µl pipette tip and remove as much supernatant as possible without aspirating beads.*
- Bind primary antibody (1 h)
  1. For each CUT&Tag and CUTAC sample, mix the primary antibody 1:50-1:100 with Antibody buffer. Resuspend beads in 25 µl per sample with gentle vortexing.
*Note: We use 1:50-1:100 antibody dilutions by default or the manufacturer’s recommended concentration for immunofluorescence. CUTAC works best using either an RNA Polymerase II CTD-phosphorylated antibody (Ser5P > Ser2P/Ser5P > Ser2P) or an α-H3K4me2 antibody. α-H3K4me3 also works but is less efficient and is depleted at enhancer sites. Several antibodies to other histone epitopes have been tested, including α-H3K4me1, α-H3K36me3, α-H3K27ac, and α-H2A.Z, but all have failed.*
  2. Place on a rotator at room temperature and incubate 1-2 h.
*Notes:*
    - *Volumes up to 50 µl will remain in the tube bottom by surface tension during rotation, avoiding the need for a quick spin before the next step.*
    - *After incubation, the tubes can be stored overnight at 4°C.*
    - *An optional negative control is performed by omitting the primary antibody.*
- Bind secondary antibody (1 h)
  1. Place tubes on the magnet stand to clear and remove and discard the supernatant.
*Note: Protein in the antibody solution improves bead adherence to the tube wall, allowing for complete removal of the liquid without dislodging the beads by doing two successive draws with a 20 µl pipettor set for maximum volume while being careful not to dislodge the beads by surface tension during the second draw.*
  2. Mix the secondary antibody 1:100 in Wash buffer and add 25 µl per sample while gently vortexing to allow the solution to dislodge the beads from the sides.
*Notes:*
    - *Calculate how much volume of diluted Antibody is necessary by multiplying the number of samples by 30 µl (which is 25 µl per sample plus overage for pipetting).*
    - *The secondary antibody step is required for CUT&Tag to increase the number of Protein A binding sites for each bound antibody. We have found that without the secondary antibody, the efficiency is very low.*
  3. Place the tubes on a rotator and rotate at room temperature for 0.5-1 h.
  4. After a quick spin (< 500 *× g* or just enough to remove the liquid from the sides of the tube), place the tubes on the magnet stand to clear and remove and discard the supernatant with two successive draws, using a 20 µl tip with the pipettor set for maximum volume.
  5. With the tubes still on the magnet stand, carefully add 500 µl of Wash buffer. The surface tension will cause the beads to slide up along the side of the tube closest to the magnet.
  6. Slowly remove 470 µl of supernatant with a 1 ml pipette tip without disturbing the beads.
*Note: To remove the supernatant, set the pipettor to 470 µl, and keep the plunger depressed while lowering the tip to the bottom. The liquid level will rise to near the top, completing the wash. Then ease off on the plunger until the liquid is withdrawn and remove the pipettor. During liquid removal, the surface tension will drag the beads down the tube. A small drop of liquid that is left behind will be removed in the next step.*
  7. After a quick spin (<500 *× g* or just enough to remove the liquid from the sides of the tube), place the tubes back into the magnet stand and remove the remaining supernatant with a 20 µl pipettor, multiple times if necessary, to remove the entire supernatant without disturbing the beads. Proceed immediately to the next step.
- Bind pA-Tn5 adapter complex (1.5 h)
  1. Mix pAG-Tn5 pre-loaded adapter complex in 300-wash buffer following the manufacturer's instructions.
  2. Pipette in 25 µl per sample of the pA-Tn5 mix while vortexing and invert by rotation to ensure that beads adhering to the sides near the top of the top are resuspended.
*Note: When using the recommended Macsimag magnet stand, dislodging the beads after resuspending in pA-Tn5 can be done by removing the plexiglass tube holder from the magnet and, with fingers on top to prevent the tubes from opening or falling out, inverting by rotating sharply a few times.*
  3. After a quick spin (<500 *× g*), place the tubes on a rotator at room temperature for 1-2 h.
  4. After incubating in the rotator, perform a quick spin and place the tubes in the magnet stand.
  5. Carefully remove the supernatant using a 20 µl pipettor twice to avoid disturbing the beads.
  6. With the tubes still on the magnet stand, add 500 µl of the 300-wash buffer.
  7. Slowly withdraw 470 µl with a 1 ml pipette tip without disturbing the beads as in Step D6.
  8. After a quick spin, place the tubes back on the magnet stand and remove and discard the supernatant with a 20 µl pipettor using multiple draws. Proceed immediately to the next step.
- Tagmentation and particle release (2.5 h) (Figure 2)
  1. Tagmentation:
    - CUT&Tag samples only: Resuspend the bead/nuclei pellet in 50 µl CUT&Tag Tagmentation buffer (10 mM mM MgCl_2_ in 300-wash buffer) while vortexing or inverting by rotation to allow the solution to dislodge most or all the beads as in Step E2.
    - CUTAC samples only: Resuspend the bead/nuclei pellet in 50 µl of either CUTAC-tag or CUTAC-hex Tagmentation buffer while vortexing or inverting by rotation to allow the solution to dislodge most or all the beads as in Step D6.
*Note: 10% 1,6-hexanediol or N,N-dimethylformamide compete for hydrophobic interactions and result in improved tethered Tn5 accessibility and library yield at the expense of slightly increased background.*
  2. After a quick spin (<500 *× g*), incubate at 37°C for 1 h (20 min for CUTAC) in a PCR cycler with a heated lid. Hold at 8°C.
  3. Place tubes on the magnet stand and remove and discard the supernatant with a 20 µl pipettor using multiple draws, then resuspend the beads in 50 µl TAPS wash buffer and invert by rotation as in Step D6.
  4. After a quick spin, place tubes on the magnet stand and remove and discard the supernatant with a 20 µl pipettor using multiple draws.
  5. Resuspend the beads in 5 µl 0.1% SDS Release solution using a fresh 20 µl pipette tip to dispense while wetting the sides of the tubes to recover the fraction of beads sticking to the sides.
*Note: Rolling the tube back and forth rapidly between thumb and forefinger while brushing the pipette tip along the sides of the tube will effectively wet the beads; follow by a quick spin to bring most of the beads to the bottom.*
  6. After a quick spin (<500 *× g*), incubate at 58°C for 1 h in a PCR cycler with a heated lid to release pA-Tn5 from the tagmented DNA.
- PCR (1 h)
  1. To the PCR tube containing the bead slurry, add 15 µl of Triton neutralization solution + 2 µl of 10 µM Universal or barcoded i5 primer + 2 µl of 10 µM uniquely barcoded i7 primers, using a different barcode for each sample. Vortex on full speed and place tubes in the metal tube holder on ice.
*Note: Indexed primers are described by Buenrostro et al. (2015). We do not recommend Nextera or NEB primers which might not anneal efficiently using this PCR protocol.*
  2. Add 25 µl NEBnext (non-hot start), vortex to mix, and perform a quick spin. Place the tubes immediately in the thermocycler and proceed immediately with the PCR.
  3. Begin the cycling program with a heated lid on the thermocycler:
    - Cycle 1: 58°C for 5 min (gap filling)
    - Cycle 2: 72°C for 5 min (gap filling)
    - Cycle 3: 98°C for 30 s
    - Cycle 4: 98°C for 10 s
    - Cycle 5: 60°C for 10 s
    - Repeat Cycles 4-5 11 times
    - 72°C for 1 min and hold at 8°C
*Notes:*
  - *To minimize the contribution of large DNA fragments and excess primers, the PCR should be performed for no more than 12-14 cycles, preferably with a 10 s 60-63°C combined annealing/extension step as described above in Step H3e.*
  - *The cycle times are based on using a conventional Peltier cycler (e.g., Bio-Rad/MJ PTC200), in which the ramping times (3°C/s) are sufficient for annealing to occur as the sample cools from 98°C to 60°C. Therefore, the use of a rapid cycler with a higher ramping rate will require either reducing the ramping time or other adjustments to assure annealing.*
  - *Do not add extra PCR cycles to see a signal by capillary gel electrophoresis (e.g., Tapestation). If there is no nucleosomal ladder for the H3K27me3 positive control, you may assume that CUT&Tag failed, but observing no signal for a sparse chromatin protein such as a transcription factor is normal, and the barcoded sample can be concentrated for mixing with the pool of barcoded samples for sequencing. Extra PCR cycles reduce the complexity of the library and may result in an unacceptably high level of PCR duplicates.*
  - *Cycle 3 (98 °C) may be extended from 30 sec to 5 min for cross-linked samples to ensure complete cross-link reversal.*
- Post-PCR cleanup (30 min)
  1. After the PCR program ends, remove tubes from the thermocycler and add 65 μl of SPRI beads (ratio of 1.3 μl of SPRI beads to 1 μl of PCR product). Mix by pipetting up and down.
  2. Let sit at room temperature 5-10 min.
  3. Place on the magnetic stand for a few minutes to allow the solution to clear.
  4. Remove and discard the supernatant.
  5. Keeping the tubes in the magnetic stand, add 200 μl of 80% ethanol.
  6. Completely remove and discard the supernatant.
  7. Repeat Steps I5-I6.
  8. Perform a quick spin and remove the remaining supernatant with a 20 μl pipette, avoiding air drying the beads by proceeding immediately to the next step.
  9. Remove from the magnet stand, add 22 µl 10 mM Tris-HCl pH 8, and vortex at full speed. Let sit for 5 min to1 h.
  10. Place on the magnet stand and allow to clear.
  11. Remove the liquid to a new 1.5 ml tube with a pipette, avoiding transfer of beads.
- Tapestation analysis (Figure 3) and DNA sequencing
  1. Determine the size distribution and concentration of libraries by capillary electrophoresis using an Agilent 4200 TapeStation with D1000 reagents or equivalent.
*Note: We use the quantification by Tapestation to estimate library concentration and dilute each library to 2 nM before pooling based on fragment molarity in the 175-1,000 bp range. The concentration 2 nM has been determined empirically as the optimal library concentration used in the HiSeq by the Fred Hutch Genomics Shared Resource.*
  2. Mix barcoded libraries to achieve equal representation as desired, aiming for a final concentration as recommended by the manufacturer. After mixing, perform an SPRI bead cleanup if needed to remove any residual PCR primers.
  3. Perform paired-end Illumina sequencing on the barcoded libraries following the manufacturer’s instructions. For maximum economy, paired-end PE25 is more than sufficient for mapping to large genomes.
*Note: Using paired-end 25* × *25 sequencing on a HiSeq 2-lane rapid run flow cell, we obtain ~300 million total mapped reads, or ~3 million per sample when there are 96 samples mixed to obtain approximately equal molarity.*
    **Figure 3.**
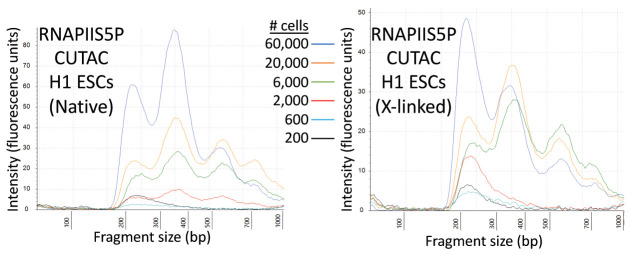
Tapestation profiles for a low-cell-number RNAPIIS5P CUTAC experiment. Tagmentation was performed for 20 min at 37°C in CUTAC-hex buffer. Representative tracks for these samples are shown in Figure 4A.

## Data analysis

1. Align paired-end reads to hg19 using Bowtie2 version 2.3.4.3 with options: --end-to-end --very-sensitive --no-unal --no-mixed --no-discordant --phred33 -I 10 -X 700. For mapping *E. coli* carry-over fragments, we also use the --no-overlap --no-dovetail options to avoid possible cross-mapping of the experimental genome to that of the carry-over *E. coli* DNA that is used for calibration. Tracks are made as bedgraph files of normalized counts, which are the fraction of total counts at each basepair scaled by the size of the hg19 genome.
*Note: To calibrate samples in a series for samples done in parallel using the same antibody, we use counts of E. coli fragments carried over with the pA-Tn5, as for an ordinary spike-in. Our sample script in* *Github* *can be used to calibrate based on either a spike-in or E. coli carry-over DNA.*
2. Our CUT&Tag Data Processing and Analysis Tutorial provides step-by-step guidance for mapping and analysis of CUT&Tag sequencing data. Most data analysis tools used for ChIP-seq data, such as bedtools, Picard, and deepTools, can be used on CUT&Tag data (Figures 3-5). Analysis tools designed specifically for CUT&RUN/Tag data include the SEACR peak caller, also available as a public web server, and CUT&RUNTools.
  **Figure 4.**
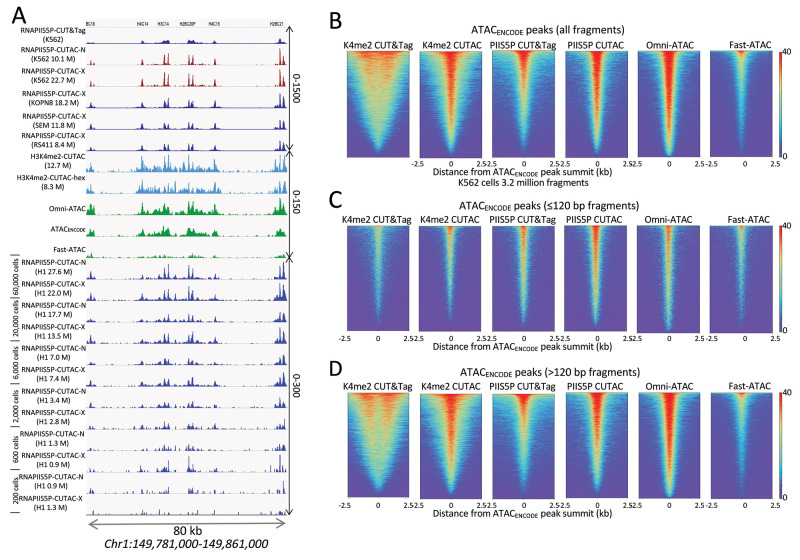
Accessible DNA corresponds to binding sites of the initiating form of RNA Polymerase II (RNAPII). **A.** Tracks show profiles of the Chromosome 1 histone gene cluster, with 12 small intronless genes expressed at high levels in all dividing cells. Whereas RNAPIIS5P CUT&Tag shows broad enrichment over each of the genes, the CUTAC protocol applied to the RNAPIIS5P epitope, either native (RNAPIIS5P CUTAC-N) or cross-linked (RNAPIIS5P CUTAC-X), yields sharp promoter delineation, better than H3K4me2 CUTAC ± 1,6-hexanediol (turquoise) or the best K562 ATAC-seq datasets (green), all downsampled to 3.2 million mapped fragments. Note the 10-fold difference in scale between RNAPIIS5P CUTAC (0-1500 and K4me2-CUTAC/ATAC (0-150). Similar results were obtained for three mixed-lineage leukemia cell lines (KOPN8, SEM, and RS411) and H1 embryonic stem cells down to ~2,000 cells. No changes were made to the protocol for low cell numbers. Numbers in parentheses are estimated library sizes in millions of mapped paired-end reads. B-D. RNAPIIS5P occupies sites of accessible chromatin in K562 cells. **B.** Left to right: K4me2 CUT&Tag, K4me2 CUTAC, RNAPIIS5P CUT&Tag, RNAPIIS5P CUTAC, Omni-ATAC, and Fast-ATAC datasets were downsampled to 3.2 million fragments and aligned over ATAC-seq peaks called using MACS2 on data generated by the ENCODE project (ATAC_ENCODE_). **C.** Same as (A) except using only subnucleosome-sized fragments (≤120 bp). CUTAC RNAPIIS5P sites are virtually indistinguishable from high-quality ATAC-seq data, directly demonstrating that ATAC-seq maps sites of the initiation form of RNA Pol II. **D.** Same as (A) except using only >120 bp fragments. ENCODE ATAC-seq fragments were downsampled to 3.2 million, ChrM (mitochondrial DNA) was removed, and MACS2 was used to call peaks. Heatmaps are centered over ENCODE ATAC-seq peak summits and ordered by occupancy over the 5 kb span displayed. Fast-ATAC is an improved version of ATAC-seq that reduces mitochondrial reads (Corces *et al.*, 2016), and Omni-ATAC is an improved version that additionally improves the signal-to-noise ratio (Corces *et al.*, 2017). ATAC_ENCODE is the current ENCODE standard (Consortium *et al.*, 2020).
  **Figure 5.**
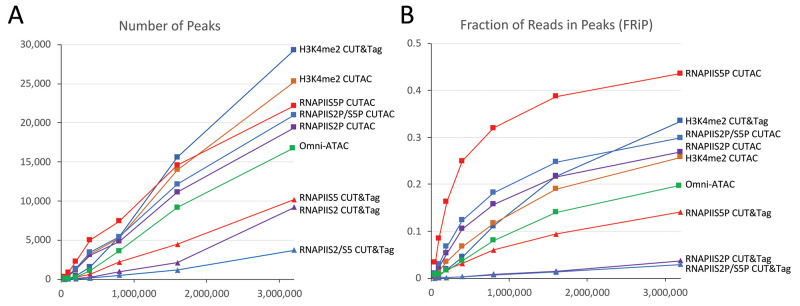
RNAPIIS5P CUTAC shows high sensitivity and specificity. Mapped fragments from the indicated K562 datasets were sampled, and peaks were called using MACS2. **A**) Number of peaks (left) and **B**) fraction of reads in peaks for CUT&Tag (triangles) and CUTAC (squares) profiles for H3K4me2, RNAPIIS5 (initiation form), RNAPIIS2P (elongation form), RNAPIIS2P/S5P, and Omni-ATAC (green). CUTAC for RNAPIIS5P shows the best sensitivity (most peaks at low sampling) and the best signal-to-noise (highest FRiP at all sampling levels). Tagmentation was for 10 min at 37°C in CUTAC-tag buffer.

## Recipes

1. Nuclei Extraction 1 (NE1) buffer
Mix 1 ml of 1M HEPES-KOH pH 7.9, 500 μl of 1 M KCl, 12.5 μl of 2 M spermidine, 500 μl of 10% Triton X-100, and 10 ml of glycerol in 38 ml dH_2_O, and add 1 Roche Complete Protease Inhibitor EDTA-Free.
2. Wash buffer
Mix 1 ml of 1 M HEPES pH 7.5, 1.5 ml of 5 M NaCl, and 12.5 μl of 2 M spermidine, bring the final volume to 50 ml with dH_2_O, and add 1 Roche Complete Protease Inhibitor EDTA-Free tablet. Store the buffer at 4°C for up to 2 days.
3. Binding buffer
Mix 200 μl of 1M HEPES-KOH pH 7.9, 100 μl of 1 M KCl, 10 μl of 1M CaCl_2_, and 10 μl 1 M MnCl_2_, and bring the final volume to 10 ml with dH_2_O. Store the buffer at 4°C for up to several months.
4. Antibody buffer
  Mix 5 µl of 200× BSA with 1 ml Wash buffer and chill on ice. BSA is present in some but not all antibody solutions, and 0.1% BSA in this buffer helps prevent bead loss during later steps.
5. 300-wash buffer
Mix 1 ml of 1 M HEPES pH 7.5, 3 ml of 5 M NaCl, and 12.5 μl of 2 M spermidine, bring the final volume to 50 ml with dH_2_O, and add 1 Roche Complete Protease Inhibitor EDTA-Free tablet. Store at 4°C for up to 2 days.
6. CUT&Tag Tagmentation buffer
Mix 1 ml of 300-wash buffer and 10 µl of 1 M MgCl_2_ (to 10 mM).
7. CUTAC Tagmentation buffer
  - CUTAC-tag: Mix 197 µl of dH_2_O, 2 µl of 1 M TAPS pH 8.5, and 1 µl of 1 M MgCl_2_ (10 mM TAPS and 5 mM MgCl_2_). Store the buffer at 4°C for up to 1 day.
  - CUTAC-hex: Mix 97 µl of dH_2_O, 100 µl of 20% (w/v) 1,6-hexanediol, 2 µl of 1 M TAPS pH 8.5, and 1 µl of 1 M MgCl_2_ (10 mM TAPS, 5 mM MgCl_2_ 10% 1,6-hexanediol). Store the buffer at 4°C for up to 1 day.
8. TAPS wash buffer
Mix 1 ml of dH_2_O, 10 µl of 1 M TAPS pH 8.5, 0.4 µl of 0.5 M EDTA (10 mM TAPS, 0.2 mM EDTA)
9. 0.1% SDS Release solution
Mix 10 µl of 10% SDS and 10 µl of 1 M TAPS pH 8.5 in 1 ml of dH_2_O
10. 0.67% Triton neutralization solution
Mix 67 µl of 10% Triton-X100 + 933 µl dH_2_O

## References

[1] Andersson R., Sandelin A. and Danko C. G.(2015). A unified architecture of transcriptional regulatory elements. Trends Genet 31(8): 426-433. 26073855 10.1016/j.tig.2015.05.007

[2] Arnold P. R., Wells A. D. and Li X. C.(2019). Diversity and Emerging Roles of Enhancer RNA in Regulation of Gene Expression and Cell Fate. Front Cell Dev Biol 7: 377. 31993419 10.3389/fcell.2019.00377 PMC6971116

[3] Auerbach R. K., Euskirchen G., Rozowsky J., Lamarre-Vincent N., Moqtaderi Z., Lefrancois P., Struhl K., Gerstein M. and Snyder M.(2009). Mapping accessible chromatin regions using Sono-Seq. Proc Natl Acad Sci U S A 106(35): 14926-14931. 19706456 10.1073/pnas.0905443106 PMC2736440

[4] Bownes M.(1990). Preferential insertion of P elements into genes expressed in the germ-line of *Drosophila melanogaster* . Mol Gen Genet 222(2-3): 457-460. 2177140 10.1007/BF00633856

[5] Buenrostro J. D., Giresi P. G., Zaba L. C., Chang H. Y. and Greenleaf W. J.(2013). Transposition of native chromatin for fast and sensitive epigenomic profiling of open chromatin, DNA-binding proteins and nucleosome position. Nat Methods 10(12): 1213-1218. 24097267 10.1038/nmeth.2688 PMC3959825

[6] Buenrostro J. D., Wu B., Litzenburger U. M., Ruff D., Gonzales M. L., Snyder M. P., Chang H. Y. and Greenleaf W. J.(2015). Single-cell chromatin accessibility reveals principles of regulatory variation. Nature 523(7561): 486-490. 26083756 10.1038/nature14590 PMC4685948

[7] Chereji R. V., Eriksson P. R., Ocampo J., Prajapati H. K. and Clark D. J.(2019). Accessibility of promoter DNA is not the primary determinant of chromatin-mediated gene regulation. Genome Res 29(12): 1985-1995. 31511305 10.1101/gr.249326.119 PMC6886500

[8] Corces M. R., Buenrostro J. D., Wu B., Greenside P. G., Chan S. M., Koenig J. L., Snyder M. P., Pritchard J. K., Kundaje A., Greenleaf W. J., Majeti R. and Chang H. Y.(2016). Lineage-specific and single-cell chromatin accessibility charts human hematopoiesis and leukemia evolution. Nat Genet 48(10): 1193-1203. 27526324 10.1038/ng.3646 PMC5042844

[9] Corces M. R., Trevino A. E., Hamilton E. G., Greenside P. G., Sinnott-Armstrong N. A., Vesuna S., Satpathy A. T., Rubin A. J., Montine K. S., Wu B., Kathiria A., Cho S. W., Mumbach M. R., Carter A. C., Kasowski M., Orloff L. A., Risca V. I., Kundaje A., Khavari P. A., Montine T. J., Greenleaf W. J. and Chang H. Y.(2017). An improved ATAC-seq protocol reduces background and enables interrogation of frozen tissues. Nat Methods 14(10): 959-962. 28846090 10.1038/nmeth.4396 PMC5623106

[10] Crawford G. E., Holt I. E., Mullikin J. C., Tai D., Blakesley R., Bouffard G., Young A., Masiello C., Green E. D., Wolfsberg T. G., Collins F. S. and National Institutes Of Health Intramural Sequencing C.(2004). Identifying gene regulatory elements by genome-wide recovery of DNase hypersensitive sites. Proc Natl Acad Sci U S A 101(4): 992-997. 14732688 10.1073/pnas.0307540100 PMC327130

[11] Dorschner M. O., Hawrylycz M., Humbert R., Wallace J. C., Shafer A., Kawamoto J., Mack J., Hall R., Goldy J., Sabo P. J., Kohli A., Li Q., McArthur M. and Stamatoyannopoulos J. A.(2004). High-throughput localization of functional elements by quantitative chromatin profiling. Nat Methods 1(3): 219-225. 15782197 10.1038/nmeth721

[12] Giresi P. G., Kim J., McDaniell R. M., Iyer V. R. and Lieb J. D.(2007). FAIRE(Formaldehyde-Assisted Isolation of Regulatory Elements) isolates active regulatory elements from human chromatin. Genome Res 17(6): 877-885. 17179217 10.1101/gr.5533506 PMC1891346

[13] Gottschling D. E.(1992). Telomere-proximal DNA in *Saccharomyces cerevisiae* is refractory to methyltransferase activity *in vivo* . Proc Natl Acad Sci U S A 89(9): 4062-4065. 1570334 10.1073/pnas.89.9.4062 PMC525632

[14] Henikoff S., Henikoff J. G., Kaya-Okur H. S. and Ahmad K.(2020). Efficient chromatin accessibility mapping in situ by nucleosome-tethered tagmentation. Elife 9: e63274. 33191916 10.7554/eLife.63274 PMC7721439

[15] Jack R. S. and Eggert H.(1990). Restriction enzymes have limited access to DNA sequences in drosophila chromosomes. EMBO J 9(8): 2603-2609. 2164473 10.1002/j.1460-2075.1990.tb07442.x PMC552293

[16] Kaya-Okur H. S., Janssens D. H., Henikoff J. G., Ahmad K. and Henikoff S.(2020). Efficient low-cost chromatin profiling with CUT&Tag. Nat Protoc 15(10): 3264-3283. 32913232 10.1038/s41596-020-0373-x PMC8318778

[17] Kaya-Okur H. S., Wu S. J., Codomo C. A., Pledger E. S., Bryson T. D., Henikoff J. G., Ahmad K. and Henikoff S.(2019). CUT&Tag for efficient epigenomic profiling of small samples and single cells. Nat Commun 10(1): 1930. 31036827 10.1038/s41467-019-09982-5 PMC6488672

[18] Consortium E. P., Moore J. E., Purcaro M. J., Pratt H. E., Epstein C. B., Shoresh N., Adrian J., Kawli T., Davis C. A., Dobin A., Kaul R., Halow J., Van Nostrand E. L., Freese P., Gorkin D. U..(2020). Expanded encyclopaedias of DNA elements in the human and mouse genomes. Nature 583(7818): 699-710. 32728249 10.1038/s41586-020-2493-4 PMC7410828

[19] Oberbeckmann E., Wolff M., Krietenstein N., Heron M., Ellins J. L., Schmid A., Krebs S., Blum H., Gerland U. and Korber P.(2019). Absolute nucleosome occupancy map for the *Saccharomyces cerevisiae* genome . Genome Res 29(12): 1996-2009. 31694866 10.1101/gr.253419.119 PMC6886505

[20] Reeves R.(1978). Nucleosome structure of Xenopus oocyte amplified ribosomal genes. Biochemistry 17(23): 4908-4916. 718864 10.1021/bi00616a008

[21] Weintraub H. and Groudine M.(1976). Chromosomal subunits in active genes have an altered conformation. Science 193(4256): 848-856. 948749 10.1126/science.948749

